# Epigenetics of Hypertensive Nephropathy

**DOI:** 10.3390/biomedicines12112622

**Published:** 2024-11-16

**Authors:** Yize Zhang, Hamidreza Arzaghi, Zhehan Ma, Yasmin Roye, Samira Musah

**Affiliations:** 1 Department of Biomedical Engineering, Pratt School of Engineering, Duke University, Durham, NC 27708, USA; 2 Center for Biomolecular and Tissue Engineering, Duke University, Durham, NC 27708, USA; 3 Division of Nephrology, Department of Medicine, Duke University School of Medicine, Durham, NC 27710, USA; 4 Department of Cell Biology, Duke University, Durham, NC 27710, USA; 5 Affiliate Faculty of the Developmental and Stem Cell Biology Program, Duke Regeneration Center, and Duke MEDx Initiative, Duke University, Durham, NC 27710, USA

**Keywords:** epigenetics of disease, hypertensive nephropathy, kidney glomerulus, kidney disease, epigenetic therapeutics

## Abstract

Hypertensive nephropathy (HN) is a leading cause of chronic kidney disease (CKD) and end-stage renal disease (ESRD), contributing to significant morbidity, mortality, and rising healthcare costs. In this review article, we explore the role of epigenetic mechanisms in HN progression and their potential therapeutic implications. We begin by examining key epigenetic modifications—DNA methylation, histone modifications, and non-coding RNAs—observed in kidney disease. Next, we discuss the underlying pathophysiology of HN and highlight current in vitro and in vivo models used to study the condition. Finally, we compare various types of HN-induced renal injury and their associated epigenetic mechanisms with those observed in other kidney injury models, drawing inferences on potential epigenetic therapies for HN. The information gathered in this work indicate that epigenetic mechanisms can drive the progression of HN by regulating key molecular signaling pathways involved in renal damage and fibrosis. The limitations of Renin–Angiotensin–Aldosterone System (RAAS) inhibitors underscore the need for alternative treatments targeting epigenetic pathways. This review emphasizes the importance of further research into the epigenetic regulation of HN to develop more effective therapies and preventive strategies. Identifying novel epigenetic markers could provide new therapeutic opportunities for managing CKD and reducing the burden of ESRD.

## 1. Introduction

Kidney diseases represent a broad spectrum of disorders that impair the organ’s structure and function, which contribute to high morbidity and mortality rates globally. More than 1 in 7 U.S. adults have CKD, resulting in an annual healthcare burden of $87.2 billion [1]. The complex etiology of kidney diseases involves a multitude of factors, including genetic predispositions, environmental exposures, and immune system dysregulation. While these conditions can present significant complication to the affected patients, they also cause substantial social and economic burdens worldwide. Among CKD patients, HN is the second leading cause of ESRD. Additionally, more than 20% of patients with hypertension eventually develop CKD, highlighting the critical interplay between these conditions [2]. Sustained high blood pressure damages the vasculature, glomerulus, and renal tubules within the nephron, leading to apoptosis of renal cells, scar tissue formation, inflammation, and compromised blood filtration function of the kidney [3]. Notably, CKD may also influence or even precede the development of hypertension, but the lack of reliable biomarkers limits the early clinical diagnosis of CKD.

Therapeutically, inhibitors of the Renin–Angiotensin–Aldosterone System (RAAS pathway) are commonly prescribed to manage HN. This treatment category includes Angiotensin-Converting Enzyme (ACE) inhibitors, Angiotensin Receptor Blockers (ARBs), Direct Renin Inhibitors (DRIs), and Aldosterone Receptor Antagonists [4]. Although these drugs can be effective in some hypertensive patients with CKD, they can interact pharmacodynamically with other medications (e.g., spironolactone and mTOR inhibitors) and induce hyperkalemia or angioedema and cause other severe side effects [5,6,7]. Patients with multiple conditions, such as hypertension and CKD, often require a complex treatment regimen, increasing the risk of side effects due to drug–drug interactions and toxicities [8].

Despite significant progress in the field, kidney disease mechanisms remain less understood. One emerging area of interest is epigenetics, which investigates modifications in gene function without alterations to the DNA sequence itself. These epigenetic modifications play crucial roles in regulating gene expression and protein function, which can profoundly impact disease development and progression. The major mechanisms of epigenetic regulation include DNA methylation, post-translational alterations to histone proteins, and non-coding RNAs, all of which have been shown to be critical in the pathogenesis and physiology of kidney disease [9,10,11]. Although various in vivo animal models and in vitro cell culture methods have been employed to elucidate some of the molecular mechanisms of HN, research on how the disease is impacted by epigenetic factors has lagged compared to other nephropathies. In this review, we describe common epigenetic events in multiple nephrotic diseases, compare the pathophysiology of HN with other CKD forms, and identify potential common and distinct underlying epigenetic mechanisms. This comprehensive review is designed to inform future research directions that could lead to more effective treatments and preventive strategies for kidney diseases.

## 2. Epigenetic Changes in Glomerular Diseases

### 2.1. DNA Methylation

DNA methylation is a well-characterized epigenetic mechanism that involves the addition of a methyl group to the cytosine base of DNA. Due to their greater stability compared to histone modifications and RNA-based regulation [12], DNA methylation can induce prolonged changes in gene expression. Previous research has revealed that the methylation of cytosine residues at the C5 position is a predominant and crucial chemical modification in mammalian genomic DNA [13,14]. Cytosine methylation within CpG islands is primarily found in promoter regions, where it generally results in the suppression of transcription, thereby playing a crucial role in gene regulation and influencing various cellular processes [15].

Multiple studies that investigated epigenetic changes in patients with kidney disease demonstrated a strong correlation between the methylation of specific loci and kidney function [16,17,18,19,20]. A recent comprehensive analysis of the epigenome in human kidney tissue samples revealed a causal relationship between the DNA methylation and pathogenesis of kidney disease [21]. Of particular interest is the proposal that prolonged ischemia reperfusion injury in kidney transplantation could lead to alterations at methylated CpG sites [22]. Mehta and colleagues [23] examined the level of DNA methylation of death-associated protein kinase (DAPK) and calcitonin related polypeptide alpha (CALCA) gene promoters in the urine of patients with kidney transplants using a quantitative methylation-specific polymerase chain reaction; the authors found a higher level of methylation in the CALCA gene promoter within transplant recipients’ urine compared to healthy controls. Additionally, there was a noticeable trend of elevated CALCA hypermethylation in the urine of patients with biopsy-confirmed acute tubular necrosis, which suggests a link between acute kidney injury (AKI) and methylation. Building on these early findings, subsequent research has shown that the levels of kallikrein (KLK1) promoter CpG methylation is significantly higher in patients with AKI compared to healthy individuals [24].

DNA methyltransferases (DNMTs) facilitate the methylation of cytosine by utilizing S-adenosyl-L-methionine as the source of the methyl groups. Recent growing evidence, such as genome-wide association studies (GWAS), suggests that DNA methylation can be a marker for diabetic nephropathy [25]. Hayashi et al. showed that the level of transcription factor Kruppel-like Factor 4 (KLF4) decreased in podocytes in DN, which elevates the binding of the DNMT1 to the nephrin promoter region and consequently reduces its expression [26,27]. A reduced nephrin expression can precede podocyte loss and is linked to the progression of kidney disease [28]. A recent study by Hishikawa et al. [29] demonstrated that knocking out the lysine acetyltransferase 5 (KAT5) gene, which plays a crucial role in DNA repair, led to elevated levels of DNMT1 and DNMT3B expression. As a result, DNA methylation in nephrin promoters increased, leading to a significant downregulation of nephrin gene expression, which, in turn, contributed to the development of DN. KLF10 is also implicated in the downregulation of nephrin expression. KLF10 directly binds to nephrin promoters and recruits DNMT1, further enhancing the methylation and suppression of nephrin gene expression [30]. Additionally, DNMT1 levels increase in response to inflammation [31]. The inhibition of DNMT1 with 5-aza-2′-deoxycytidine (5-Aza) caused regulatory T cells to co-express CD4 and CD25 (CD4^+^CD25^+^), leading to significantly improved outcomes in mice with diabetic kidney disease. Intriguingly, reducing DNMT1 expression through RNA interference caused a significant demethylation of cytosine in mTOR-negative regulators, leading to decreased mTOR activity [31]. While reducing the DNMT1 expression has shown effects on mTOR-negative regulators and decreased mTOR activity in mice, similar mechanisms have also been observed in human studies. For instance, DNMT1 knockdown in human kidney cells, such as renal tubular epithelial cells and podocytes, enabled the restoration of phosphatase and tensin homolog (PTEN) expression, a key negative regulator of the mTOR pathway. This result was accompanied by a decrease in mTOR signaling activity, indicating that DNMT1 plays a similar role in the kidney cells examined in the study [32].

DNA methylation can be reversed by actions of the ten-eleven translocation proteins (TET), which are essential to the physiological and pathophysiological processes in the kidney. Knockout studies showed that TET2 could be a promising target for treating ischemia reperfusion injury [33]. Furthermore, TET2 plays a crucial role in promoting the DNA demethylation and subsequent gene expression essential for podocyte structure and function. For example, TET2-driven pathways, including the demethylation of podocyte-specific genes like Kirre Like Nephrin Family Adhesion Molecule 1 (KIRREL1), and Nephrotic syndrome 1 (NPHS1), offer protective effects in models of kidney injury, helping to maintain podocyte integrity and reduce cell damage under stress [34]. Interestingly, in urine samples from patients with hypertension, both DNMT and TET enzyme levels were elevated, which is hypothesized to be due to DNA damage and repair mechanisms [35]. However, Liu et al. [36] demonstrated that administering a combination of anti-Dnmt3a and anti-Tet3 GapmeRs (Gapmer antisense oligonucleotides) to the renal outer medulla of Dahl Salt-Sensitive (DS) rats on a high salt diet significantly reduced hypertension. In contrast, anti-Dnmt3a alone did not manage hypertension as effectively as the combined treatment of anti-Dnmt3a and anti-Tet3. Although further research is needed to fully understand the effects of DNA methylation and demethylation in hypertension, studies have shown that demethylation can cause hypertension in tissues beyond the kidneys, such as the hypothalamus [37].

### 2.2. Histone Modifications

Histones are essential proteins that form the core of nucleosomes, which help compact DNA into the structured form found in chromosomes. The N-terminal tails of histones are subject to various post-translational modifications, such as acetylation, methylation, phosphorylation, and ubiquitination. Histone modifications can drastically transform chromatin function by altering the charges of amino acids, which weakens the interaction between histones and DNA. Such weakened interactions facilitate the transition between open (euchromatin) and closed (heterochromatin) chromatin states, thereby regulating the accessibility of chromatin and subsequent transcription. There is substantial evidence that such changes in chromatin structure and accessibility play a critical role in the progression of kidney complications.

Among these modifications, histone methylation—involving the transfer of methyl groups to lysine and arginine residues—plays a pivotal role in podocyte survival and function by influencing the structure of the slit diaphragm. Specifically, the histone methyltransferase Enhancer of Zeste Homolog 2 (EZH2) mediates the trimethylation of lysine residue 27 on histone protein H3 (H3K27me3). This methylation is crucial for maintaining normal podocyte function. The deletion of EZH2 from podocytes decreases the levels of H3K27me3, which subsequently increases the susceptibility of mice to glomerular disease [38]. Inhibiting lysine demethylase 6b (KDM6B) and 6a (KDM6A) led to elevated H3K27me3 levels in podocytes, which mitigated glomerular disease specifically in diabetic nephropathy models (db/db mice) and in mouse models of Adriamycin-induced nephrotoxicity. Additionally, human kidney tissue samples from patients with diabetic nephropathy and focal segmental glomerulosclerosis showed similar epigenetic alterations [38]. A recent study by Lin and colleagues [39] demonstrated that hyperglycemia amplifies a positive feedback loop between KLF10 and KDM6A and discovered that KLF10 recruits DNMT1 to the nephrin promoter, thus inhibiting its expression. Knocking out either KDM6A or KLF10 in mice significantly reduced diabetes-induced proteinuria and kidney damage. However, it is important to consider the context of these studies before drawing any conclusions, as more recent findings have presented some contrasting results. A recent study by Yu et al. [40] showed that KDM6B plays a necessary role in the inhibition of inflammation and the profibrotic response. They demonstrated significant renal protection and regeneration in mouse models of AKI induced by ischemia/reperfusion and folic acid (FA) toxicity by upregulating KDM6B expression, which promoted tubular epithelial cell survival and regeneration. Blocking KDM6B activity worsened renal dysfunction and increased cellular apoptosis, while its activation enhanced tubular cell proliferation and suppressed inflammatory and profibrotic pathways.

Histone acetylation is another crucial post-translational modification important for gene regulation; it involves the addition of an acetyl group to the lysine residues on histone proteins, which causes the relaxation of the chromatin structures and enhances the accessibility of the DNA to transcription factors and other regulatory proteins. Histone acetylation is a key epigenetic modification that significantly contributes to the pathogenesis of CKD [41]. This histone modification can be reversed with histone deacetylases (HDACs), which present a compelling target for therapeutic intervention due to their abnormal activity being linked to heightened fibrosis and inflammation, which are hallmarks of diabetic nephropathy [42]. For example, a recent study by Wang et al. [43] showed that HDAC3 is a critical regulator of inflammation and fibrosis in mice. However, since these enzymes’ effects are global, their expression and localization should be investigated in physiological and pathophysiological contexts. For example, Hyndman and colleagues [44] showed that, when the kidneys undergo ischemia reperfusion, there is an activation of histone deacetylases HDACs, which is characterized by isoform-specific expression patterns that highlight the unique roles of each variant. Hyndman and colleagues noted that ischemia-reperfusion injury activates HDACs, leading to fibrosis. However, treatment with HDAC inhibitors resulted in a reduction of fibrosis markers and a decrease in Ki-67-positive nuclei, indicating a reduction in proximal tubule proliferation and suggesting a complex role for HDACs in balancing fibrosis and the tissue repair mechanism.

In addition to the aforementioned histone modifications, various studies have highlighted the importance of epigenetic markers in different kidney diseases. There is evidence indicating significant alterations in the level of H3K4me3 of fc receptor like 4 (FCRL4) and galactokinase 2 (GALK2) in peripheral blood mononuclear cells of IgA nephropathy patients [45]. The knockout of KDM6A in tubular cells results in increased levels of H3K4me3, which contributes to elevated blood pressure and hypertension [46]. An analysis revealed that the levels of Na-K-2Cl cotransporter and Na-Cl cotransporters were significantly elevated, leading to reduced sodium excretion in KDM6A-cKO mice. Additionally, the increased expression of aquaporin 2 (AQP2) in these mice suggests a role in enhancing water reabsorption, further contributing to the observed high blood pressure. Previous studies have shown that high glucose levels in renal tubular epithelial cells can cause an epigenetic increase in connective tissue growth factor (CTGF) levels, which leads to elevated levels of acetylated histone H3 (AcH3) and trimethylated H3K4 (H3K4Me3) in the CTGF promoter region and decreased levels of dimethylated H3K9 (H3K9Me2). Under high-glucose conditions, ASH2-like protein (ASH2L) and H3K4me3 are drawn to the promoter regions of ADAM metallopeptidase domain 17 (ADAM17) and homeodomain interacting protein kinase 2 (HIPK2). This recruitment triggers irregular RNA transcription, which subsequently activates the neurogenic locus notch homolog protein 1 (Notch1) signaling pathway. This cascade of events plays a significant role in promoting fibrosis and inflammation associated with diabetic nephropathy [47].

Other forms of histone modification are implicated in the development of kidney disease. For example, histone lysine crotonylation (Kcr) is a widely occurring post-translational modification, akin to acetylation (Kac). Despite its prevalence, the specific functions of Kcr in kidney disease have yet to be fully understood. Nevertheless, a recent study by Li and co-workers [48] suggested that increasing H3K9 crotonylation (H3K9cr) worsens kidney fibrosis. This modification’s level, as highlighted in the study by Li and co-workers, can be adjusted by acyl-CoA synthetase short-chain family member 2 (ACSS2), which further regulates interleukin-1 beta (IL-1β)-mediated macrophage activation and the senescence of tubular cells.

There is growing evidence that links histone lactylation with CKD; Wang et al. [49] uncovered a significant role for 6-phosphofructo-2-kinase/fructose-2,6-biphosphatase 3 (PFKFB3) in CKD. Their research revealed a marked upregulation of this gene specifically in proximal tubule cells of both human patients and mouse models. Strikingly, the areas with an elevated PFKFB3 expression and increased lactate production were directly associated with severe fibrosis and inflammation. The lactylation of Histone H4 lysine 12 (H4K12) significantly boosted the transcriptional activation of genes within the nuclear factor-κB (NF-κB) signaling pathway, amplifying its regulatory effects. As previously mentioned, similar to methylation and acetylation, there are also contrasting views on the effects of histone lactylation. Although it was initially believed to solely promote gene transcription, emerging evidence suggests a more complex role; a recent study showed that histone lactylation at H3K18 can reduce the transcriptional activation of Retinoic acid receptor gamma (RARγ), a receptor that plays a critical role in cell differentiation, proliferation, and apoptosis [48,50].

### 2.3. Non-Coding RNA

Non-coding RNAs (ncRNAs) are a diverse group of RNA molecules that do not encode proteins but play crucial roles in regulating gene expression and maintaining cellular functions. Unlike messenger RNAs (mRNAs) that serve as templates for protein synthesis, ncRNAs function in various biological processes, including chromatin remodeling, transcriptional regulation, RNA splicing, and translation. They are broadly categorized into small ncRNAs, like microRNAs (miRNAs) and small interfering RNAs (siRNAs), and long non-coding RNAs (lncRNAs).

It has been demonstrated that lncRNAs are involved in the progression of diabetic nephropathy. The expression of DLX6-AS1 in patients with diabetic nephropathy was shown to correlate with the severity of albuminuria [51]. DLX6-AS1 is an lncRNA that regulates gene expression and has been implicated in various diseases, including cancer and diabetic nephropathy, where its elevated expression contributes to cellular damage and inflammation by modulating pathways such as glycogen synthase kinase-3 beta (GSK-3β) through microRNAs like miR-346. Cultured podocytes with a higher DLX6-AS1 expression exhibited a damaged phenotype and inflammatory responses through the miR-346-mediated regulation of the GSK-3β pathway, contributing to the progression of diabetic nephropathy [51].

The role of lncRNAs has been observed in other kidney complications, such as AKI. A recent study by Xue et al. [52] demonstrated that 66 out of 218 patients with severe AKI exhibited higher levels of nuclear paraspeckle assembly transcript 1 (NEAT1) in urinary sediment cells. The overexpression of NEAT1 via TLR4/NF-κB signaling activates protein kinase C, leading to the activation of the NLR family pyrin domain containing 3 (NLRP3) inflammasome. This pro-inflammatory role of lncRNAs in kidney disease have been observed in other cell types in the nephron. For example, a recent study showed that a high-glucose environment causes glomerular endothelial cells to upregulate lncRNA-carboxylesterase 1 pseudogene 1 (CES1P1), which subsequently inhibits miR-214–3p. This inhibition increases the levels of inflammatory cytokines such as IL-17, IκB, NF-κB, and IL-6 [53]. In another example, lncRNA-Snhg1 was shown to bind directly to miR-27b in vitro, blocking its interaction with KDM6B mRNA. This interference led to the increased expression of inflammatory cytokines and the proliferation of mesangial cells, even in low-glucose environments [54]. Therefore, lncRNAs not only contribute to kidney disease pathogenesis but also interacts with other epigenetic modifications—such as histone modifications and DNA/RNA methylation and acetylation—to influence disease mechanisms [55]. For example, the hypermethylation of the maternally expressed 3 (MEG3) promoter by DNMT1 led to the blocking of its expression and promotion of kidney cell fibrosis via the miR-185/DNMT1/MEG3 pathway [56]. Numerous studies have demonstrated the interactions between lncRNA and histone modifications [55,57,58,59]. For example, under high-glucose conditions, lncRNA-Dlx6os1 recruits an EZH2, which targets the H3K27 histone. This recruitment accelerates the progression of diabetic nephropathy by epigenetically repressing SRY-box transcription factor 6 (SOX6) [60]. Together, these findings emphasize the pivotal role of lncRNAs in kidney disease progression, illustrating how their interactions with other epigenetic modifications can contribute to disease mechanisms and highlighting potential targets for therapeutic intervention.

## 3. Methodological Approaches for Investigating HN

### 3.1. In Vivo Models

One of the most common standards for researching HN is the use of animal models, particularly rodents, due to the ease of genetic manipulation and substantial amount of research and existing data for physiological extrapolation to humans [61]. There is a wide range of animal hypertension models, developed through genetic, pharmacological, and surgical methods, which have been instrumental in understanding the development of HN in vivo.

#### 3.1.1. Genetic Models

Spontaneously hypertensive rats (SHRs) are a well-established genetic model for studying hypertension; its early history traces back to Dr. Kozo Okamoto and Dr. Kyuzo Aoki in the early 1960s through selective breeding of Wistar–Kyoto rats with inherent high blood pressure [62]. SHRs naturally develop elevated blood pressure around 4–5 weeks of age, and, by 12–15 weeks old, their blood pressure is significantly higher compared to normotensive control rats. In the kidneys, SHRs exhibit early-onset proteinuria, which progresses to glomerulosclerosis, tubular atrophy, and interstitial fibrosis [63].

Mihailović-Stanojević et al. [64] utilized the SHR model to study the effects of losartan on the progression of adriamycin-induced nephropathy under hypertensive conditions. They found that losartan restored renal function, reduced proteinuria, and even lowered blood pressure in SHR. Another study investigated the protective effect of the combination of Gedan Jiangya Decoction (GJD) and captopril on hypertensive kidney injury in SHRs and showed that the combination therapy reduced fibrosis, tissue damage, and the expression of hypertension-related renal vascular markers [65]. A transcriptomic analysis of renal tissue from SHRs with HN also indicated upregulation of genes related to inflammation and dysregulation of lipid metabolism [66].

In the 1960s, another selectively bred rat model (i.e., the DS Rat model) for studying HN was developed by Lewis Dahl [67]. Unlike SHRs, hypertension in DS rats is induced by a high-salt diet and therefore the onset of hypertension can be controlled. DS rats have increased renal histopathological damage, decreased creatinine clearance, accumulation of Transforming growth factor beta-1 (TGF-β1), and recruitment of M1 proinflammatory macrophages in the kidney [68,69,70]. A more recent study used the DS Rat model to study the traditional Chinese medicine QiShenYiQi, which has been demonstrated to ameliorate renal interstitial fibrosis and collagen deposition in DS-induced HN [71]. Johnson et al. demonstrated the effect of loss of Rho guanine nucleotide exchange factor 11 (Arhgef11) on the reduction of proteinuria symptoms using the DS rat model [72]. The DS rat model has also been used to study the effect of epigallocatechin-3-gallate (EGCG), one active catechin in green tea, on alleviating renal injury through antioxidant and anti-inflammatory effects [73].

Besides HN, systematic hypertension has also been shown to induce injury in other organs. In the brain, hypertension induce hypertensive brain damage, and SHRs have been wildly used for studying vascular brain disorders [74]. Additionally, hypertension in genetic rat model has also induced pulmonary hypertension which leads to condition such as pulmonary venous sphincter hypertrophy [75]. Despite the predictability and effectiveness of the SHR and DS rat models, their genetic homogeneity and oversimplification of the causes of hypertension and HN limit their relevance to human physiology and disease mechanisms.

#### 3.1.2. Drug-Induced Models

Another way to induce hypertension and HN in animal models is through the use of bioactive compounds. Angiotensin II (Ang II), a well-known vasoconstrictor, has been used to induce hypertension in rodents through continuous infusion [76]. Several studies have utilized the Ang II hypertension model to investigate various therapeutic strategies by targeting the Ang II/ RAAS system. For instance, Liu et al. [77] demonstrated that overexpression of suppressor of mothers against decapentaplegic 7 (SMAD7) (a downstream inhibitor of both the Ang II and NF-κB pathways) in mice could protect against Ang II-mediated nephropathy and halt renal injury after 14 days of Ang II infusion. Additionally, the Ang II model has been combined with genome editing in mice to illustrate how CRISPR knockout of soluble (pro)renin receptor (sPRR) reduces the activation of the renin–angiotensin system (RAS) and, therefore, inflammation and fibrosis in the kidney [78]. Conversely, ACE2 knockout significantly exacerbated hypertension-induced renal injury, as indicated by increased serum creatinine levels and progressive renal inflammation and fibrosis [79].

Another commonly used pharmacological method for inducing hypertension in rodents is the Deoxycorticosterone acetate (DOCA) (a mineralocorticoid) salt model, which involves the administration of DOCA combined with a high-salt diet. This approach leads to fluid retention in blood vessels, increased blood volume, and elevated blood pressure due to enhanced sodium reabsorption. The peroxisome proliferator-activated receptor gamma (PPARγ) agonist Rosiglitazone [80], Sildenafil [81], and ACE inhibitors [82] have been studied in the DOCA salt model for their renal protective effects. The ACE inhibitor omapatrilat decreases mesangial collagen deposition and reduces inflammatory marker levels in DOCA salt rats [83]. Similarly, sildenafil attenuates tubulointerstitial fibrosis and glomerulosclerosis while also reducing cell apoptosis in DOCA salt rats [81].

However, both the Ang II and DOCA pharmacological models have some significant limitations. Specifically, hypertension in these models is induced under supraphysiological conditions, such as continuous infusion of large doses of Ang II, or is limited to specific types of hypertensions, such as salt-induced hypertension and mineralocorticoids excess in the DOCA salt model. These conditions lead to rapid injuries in hypertensive kidneys, which do not accurately represent the complex causes of HN in vivo. More importantly, these models do not recapitulate the gradual progression of HN typically resulting from chronic hypertension.

#### 3.1.3. Surgical Models

There are three well-established surgical models for studying hypertension and HN, and they all involve constricting the renal artery and blood flow into the kidney or removing parts or all of a kidney. The Two-Kidney, One-Clip (2K1C) model, first described by Harry Goldblatt and colleagues in the 1930s [84], involves partially constricting one renal artery using a clip, which reduces blood flow to one of the two kidneys. The reduced blood flow stimulates the release of renin, leading to the activation of the RAAS system, which has been shown to cause glomerular sclerosis and subsequent loss of filtration function in the non-clipped kidney [85,86]. Similar to the 2K1C model, the One-Kidney, One-Clip (1K1C) model constricts the renal artery in one kidney but removes the other kidney. Both of these surgical models share a similar pathophysiological mechanism, but, due to the lack of a compensatory kidney, the 1K1C model typically results in more severe hypertension and kidney injury [87,88] and is less commonly used in the study of HN.

Recent HN research has utilized the 2K1C model to understand the role of the Ang II Type 1A Receptor (AT1R) in the disease. Alawi et al. showed that hypertension induced by 2K1C model decreases the expression of ACE2 and nprilysin (NEP), and deletion of AT1R attenuate the severity of albuminuria and hypertension in 2K1C rats [89,90]. Another research group showed that the use of the bioactive flavonoid glycoside vaccarin reduces expression of fibrosis markers (collagen-I, collagen-III, and fibronectin) and inflammatory cytokines (TNF-a, IL-1β, and IL-6) in the right kidney of 2K1C rats [91].

The partial nephrectomy (PNx) is common in patients who have had a kidney tumor removed. The 5/6 nephrectomy (removal of one entire kidney and 2/3 of the other) has been used as a model in rodents to mimic renal failure. Rodents subjected to the procedure exhibit multiple features including hypertension, a reduced number of nephrons, hyperfiltration in the remaining glomeruli, glomerular hypertrophy, mesangial expansion, increased glomerular collagen deposition, and an increase in activity of the RAAS system [92,93,94]. Additionally, the surgical reduction of renal mass often triggers inflammation responses, further damaging kidney tissue [95].

In recent studies on HN, polysulfate pentosan (PPS), a sulfated oligosaccharide, has been tested in 5/6 nephrectomy rats and demonstrated to prevent glomerular hyperfiltration and hypertrophy [96]. Therapeutics targeting the inflammatory responses in 5/6 nephrectomy through knockdown of periostin show decreased production of proinflammatory cytokines tumor necrosis factor alpha (TNF-a) and IL6, which protects renal tissue from further injury [95]. Additional independent studies also show that 5/6 nephrectomy induces ferroptosis, a less understood mechanism of cell death in the kidney, which leads to fibrosis in the tissue; in addition, study of kidney anemia using 5/6 nephrectomy have underscored the importance of iron metabolism in kidney fibrosis [97,98].

Despite the rapid and drastic pathological responses that can be useful for studying acute injuries, the invasive surgical process can introduce issues, such as inflammation and surgical stress, which potentially complicates biological interpretation of disease onset and mechanisms in HN models. Additionally, the surgical models of HN heavily rely on compensatory hypertrophy and hyperfiltration of the remaining nephrons, which may lead to model-specific artifacts or injury patterns not seen in HN without nephron loss.

Overall, animal (mostly rodent) models of HN provide an established means of studying the disease at the organismal level. Comparative studies, such as the one by Gutsol et al. [99], highlight the relevance of hypertensive nephrosclerosis in rodent models to human pathology and underscore the translational value of these models. However, these animal models are limited in their ability to accurately represent human physiology and disease mechanisms, which limits their translational potential.

### 3.2. In Vitro Models

#### 3.2.1. 2D Cell Culture

In vitro cell culture systems can offer significant advantages over animal models, particularly in their ability to include human or patient-derived cell populations, thus potentially providing more relevant insights into human biology and disease mechanisms. However, the rigid and sometimes nonphysiologically relevant nature of traditional tissue culture plates make them challenging to apply when trying to simulate the biophysical as well as molecular aspects of hypertension, which include dynamic high fluid flow and shear stress, cyclic mechanical stretch, tissue–tissue interfaces, and cellular crosstalk. In 2006, Friedrich et al. reported a study involving the use of custom-designed two-chamber fluid flow system lined with immortalized mouse podocytes; the authors demonstrated in vitro that podocytes are sensitive to high fluidic shear stress, which causes the cells to undergo cytoskeletal rearrangement [100].

The Flexcell system, a well-established 2D cell culture bioreactor, allows for precise control of mechanical stretch and fluidic flow in cell cultures using elastic, stretchable silicone elastomers or flow chambers. Several studies have used mouse primary cells cultured in the Flexcell strain system to simulate the effects of HN on specific cell populations. For example, one study found that applying 10–20% cyclic stretch to mouse mesangial cells significantly upregulated the SMAD6 gene [101]. Another study applied 15% static mechanical strain to mouse podocytes which resulted in chronic podocyte injury induced by activated ras homolog family member A (RhoA) and ras-related C3 botulinum toxin substrate 1 (Rac-1) [102]. Beyond findings related to cell mechanosensitive pathways, the Flexcell 2D culture system has also elicited effects similar to HN-induced injury in vivo, such as Ang II-AT1 activation-induced podocyte injury and the loss of the key slit diaphragm protein nephrin [103,104].

In addition to the commercialized Flexcell system, custom-designed bioreactors or loading systems have been used to study the effects of fluidic shear stress on renal tubular cells and podocytes. One study employed immortalized human proximal tubular cells on custom-designed gelatin-coated plastic slides using a peristaltic pump to apply a high flow rate of 90 μL/min. This experimental parameter resulted in increased secretion of inflammatory cytokines, resembling responses seen in renal tubules in salt-induced hypertensive rats [105].

While simpler 2D bioreactor systems have successfully recapitulated some aspects of hypertension-induced kidney injury observed in vivo, these methods primarily focus on monocultures of single-cell populations and lack the complexity of in vivo systems, including cell–cell interactions. Furthermore, these studies typically use extremely rigid plastic surfaces, which do not represent the molecular makeup and structural integrity of the native extracellular matrix (ECM). Many of the limitations mentioned above can be addressed using more advanced in vitro models, such as organ-on-chip and organoid systems.

#### 3.2.2. Organoids

An organoid is a multicellular cell mass that mimics some aspects of the target organ’s tissue structure and function. Kidney organoids are typically generated by differentiation of human pluripotent stem cells into nephron progenitor cells, which then self-organize into complex structures resembling segments of the nephrons [106]. Takasato et al. [107] demonstrated that kidney organoids derived from human induced pluripotent stem (iPS) cells contain multiple cell lineages, including podocytes, proximal tubules, and distal tubules, effectively serving as a model system for studying human nephrogenesis.

Despite significant advancements, no method currently exists to model HN using organoids alone. Implementing organoids as a model for studying HN could involve the use of hypertension-inducing drugs, such as Ang II, to promote vasoconstriction and stimulate pro-fibrotic pathways. Additionally, applying high mechanical stretch at levels relevant to HN could help recapitulate the effects of elevated blood pressure on kidney tissue, particularly on glomerular and tubular cells. Finally, if the issue of limited vascularization in organoids can be addressed, increasing blood flow rates within the organoid’s vascular system via a microfluidic device could create a highly physiologically relevant model for studying the effects of HN on kidney tissue structures.

In addition, key features exhibited by kidney organoids, such as segmental organization and active cell–ECM interactions, make them valuable for studying the cellular and molecular mechanisms underlying HN. A key pathological feature of hypertension-induced kidney injury is the interstitial fibrosis observed in HN, characterized by excessive ECM deposition due to the activation of renal fibroblasts in response to systemic disturbances in oxidative stress, inflammation, and the RAAS [108]. While 2D cell cultures are limited in studying cell-type specific ECM deposition and fibrosis formation, organoids and organs-on-chips systems could provide the desired cell populations and tissue organization, as well as ECM structures, to study fibrotic processes. Preliminary efforts allowed for the analysis of pro-fibrotic responses in multiple kidney cell types and their interactions with fibrotic tissue, as demonstrated in a recent study on the effects of SARS-CoV-2 on kidney fibrosis using human iPS-cell-derived organoids [109].

#### 3.2.3. Organ on Chip

Kidney-on-chip technology has emerged as a more physiologically relevant in vitro platform for studying HN, offering an advanced approach to mimic in vivo conditions within a controlled multicellular environment. This technology enables precise control of multicellular environments, closely replicating the complex in vivo conditions of the kidney—in the case of the glomerular filtration barrier, which consists of a podocyte layer and an endothelial cell layer (which can be represented by the two cell chambers in the chip), the basement membrane can be mimicked using a semi-permeable membrane that separates the two chambers. Various segments of the nephron have been successfully modeled using organs-on-chips systems, including proximal tubule-on-a-chip [110], collecting-duct-on-a-chip [111], distal tubule-on-a-chip [112], glomerulus-on-a-chip [113,114,115], and nephron-on-a-chip [116], and each provides a unique platform to investigate specific renal functions and pathologies. Among these, the glomerulus-on-a-chip system is particularly significant for studying HN as it directly models the site of filtration where hypertensive damage often occurs.

In 2016, Zhou et al. [117] developed a polydimethylsiloxane-based dual-channel glomerulus-on-a-chip model featuring a porous polycarbonate membrane to simulate the glomerular basement membrane between glomerular endothelial cells and podocytes. They simulated a hypertensive environment in the glomerulus by applying perfusion flow rates of 5, 10, and 15 μL/min, observing dynamic alterations in the cytoskeletal architecture of podocytes and reduced expression of the podocyte markers nephrin and podocin. A recent study using a similar chip design with air pressure-controlled flow demonstrated flow-dependent toxicological responses to doxorubicin and puromycin in podocytes [118].

In 2017, Musah et al. developed a more physiologically relevant glomerulus-on-a-chip model using iPS-cell-derived podocytes and primary glomerular microvascular endothelial cells, later incorporating iPS-cell-derived glomerular endothelial cells [113,114,115]. This model recapitulated the filtration barrier function of the glomerulus and its response to adriamycin-induced injuries. The study also uncovered the effect of cyclic strain on improving podocyte differentiation, and glomerular tissue formation and function, as demonstrated by the increased podocyte slit diaphragm protein marker (nephrin) expression levels and increase in vascular endothelial growth factor A (VEGF-A) secretion (which promotes glomerular vascular patterning). Such models can be easily modified to create a hypertensive environment for studying HN by modulating the fluid flow rate in the system and applying cyclic mechanical stretch to a hypertension-relevant level or frequency. Following the Musah et al. kidney chip model, Mou et al. applied a biomimetic ultrathin silk fibroin-based membrane to engineer a glomerulus-on-a-chip system with tissue–tissue interfaces exhibiting in vivo-like proximity and crosstalk between podocytes and endothelial cells that enabled induction of fenestrated endothelium from iPS-cell-derived glomerular endothelial cells [115]. While studies on renal tubules-on-a-chip systems under hypertension are underexplored, the ability to control fluid flow rates and apply cyclic stretch can be extended to existing tubules-on-a-chip systems to better understand how tubular cells respond to external biophysical stress and their mechanobiological and molecular alterations in a hypertensive environment, or to study the correlation between ECM remodeling in hypertensive nephropathy-induced tubulointerstitial fibrosis and the mechanical properties of the basement membrane in future research.

Both organ-on-chip and organoid models have their respective strengths and weaknesses in modeling HN and other kidney diseases. Organ chip systems provide precise control over the microenvironment and fluidic conditions, enabling better simulation of physiological processes, such as filtration and shear stress. However, most kidney-on-chip platforms utilize a bilayer or trilayer setup with 2–3 cell types in a primarily 2D culture, which lacks the complexity of the in vivo environment. Conversely, organoids, as 3D structures, incorporate a wider range of cell types found in vivo. However, organoids often form dense, heterogeneous cell clusters that exhibit immature or fetal-like tissue characteristics and off-target cell types, which limit reproducibility and analyses requiring tissue-specific patterning and function. The lack of spatiotemporal control in organoids hinders their ability to model dynamic biological processes such as modeling the blood filtration function and tubular reabsorption and secretion accurately since they typically lack proper vasculature and blood flow. Consequently, current kidney organoid technologies do not replicate the precise cell–environment interactions that are more effectively represented in organ-on-chip models with dedicated fluid compartments that can be selectively perfused [119].

Recent developments have combined kidney organoids with organ-on-chip microfluidic systems to create an organoid-on-chip platform. This combination has greatly enhanced the study of HN by simulating the dynamic microenvironment of the kidney, including vascularization, mechanical forces, and nutrient perfusion. This integration allows for more physiologically relevant modeling of renal conditions, enabling detailed exploration of how hypertension affects kidney function. Key studies have demonstrated the utility of these systems to mimic blood flow, study mechanosensing mechanisms, and investigate kidney–vasculature interactions. These innovations offer powerful tools for understanding disease mechanisms and developing targeted therapies, potentially bridging the gap between traditional in vitro systems and in vivo strategies to promote translational research and related clinical applications [120,121,122,123,124].

#### 3.2.4. Computational Models

Computational modeling tools have increasingly become accessible to nearly every subfield of biology and medicine. The field of epigenetics has largely benefited from the development of deep sequencing techniques which enable robust segmentation of critical pathways in human development, homeostasis, and pathology. In 2007, Roberston et al. reported their study establishing the Chromatin Immunoprecipitation Sequencing (ChIP-seq) method used to identify DNA–protein binding sites [125]. Since then, next-generation sequencing methods have been developed, such as the assay for transposase-accessible chromatin with sequencing (ATAC-seq) method [126], which can assess genome-wide chromatin accessibility and subsequently relate chromatin packaging with other factors that influence gene expression. Research and clinical nephrology are poised to benefit from these high-resolution techniques, especially spatial transcriptomics and single-cell RNA sequencing, that can overcome the cellular complexity of the kidney and uncover gene-expression-level changes in health and disease. In 2023, Huyan et al. employed single-cell RNA-seq to study early-stage hypertensive kidney injury in rats [127]. Across the 10,000 single cells analyzed, the authors found that there was no loss of kidney cell types but rather a significant influx of immune cell types (neutrophils and natural killer cells) and reduction in endothelial and smooth muscle cells, indicating vascular injury. After documenting the gene expression changes associated with distinct regions of the nephron (loops of Henle, proximal tubule, glomerulus and vasculature, the distal convoluted tubule, and collecting duct), the authors concluded that the most notable HN-associated damage occurred at the glomerulus. Furthermore, the authors described the role of intercellular communication on glomerular inflammation in early-stage HN: elevated expression of the chemokines atypical chemokine receptor 3 (Ackr3) and C-X-C motif chemokine ligand 12 (Cxcl12) that originates from mesangial cells and extends to the podocytes and endothelial cells that incites vascular damage, thereby suggesting that the mesangium is a dominant player in contributing to glomerular loss due to hypertensive injury. This work lays the foundation to identify expression patterns at early stages of hypertension to potentially develop interventions that protect against kidney injury. 

Computational modeling tools can provide detailed insights when used as individual methods, but, when integrated with in vitro disease modeling, a comprehensive profile of developmental and functional characteristics of tissues, organs, and even whole organisms could be uncovered. In 2024 alone, several noteworthy multi-omics studies related to the kidney and HN were reported. One study by Adedini et al. used single-cell sequencing in combination with ATAC-Seq to uncover the role of mineralocorticoids in the induction of HN [128]. Haug et al. used adult male kidney biopsies to produce a genomic map that distinguishes the epigenomic landscapes of the kidney’s cortex and medulla [129]. The work encompasses gene expression (RNA sequencing), chromatin accessibility (ATAC-seq), and chromatin conformation (Hi-C) that was validated by spatial transcriptomics and immunohistochemistry. The authors were able to (re)assign cortex- or medulla-specific gene expression signatures in the Genotype-Tissue Expression (GTEx) Project database, where medulla samples are scarce, and provided data for the Human Protein Atlas, which previously lacked transcriptomic data for the medulla, despite being a critical site of diseases such as developmental defects and acute kidney injury. Shortly thereafter, Xu et al. published their investigations of HN using computational methods [130] in which the authors generated gene expression prediction models to analyze the transcriptome-wide association studies in 700 human kidneys. Among several dataset inputs, the authors used the GTEx database to predict the components of gene expression that directly act through genetic influence (barring noise from temporal and environmental factors) and prioritized cell- and tissue-types that relate to blood pressure. Their model applied the Prediction Using Models Informed by Chromatin conformations and Epigenomics (PUMICE) algorithm [131] to the kidney, that uniquely utilizes both 3D genomic and epigenomic data to improve power and accuracy in gene expression models and transcriptome-wide association studies, and then integrated the algorithm with RNA-seq transcriptome data. The authors detected approximately 24% increase in predictive model generation compared to PrediXcan—one of the most widely used single-tissue elastic net models. With their model, the authors uncovered 889 unique kidney genes with significant association with at least one blood pressure summary statistics (systolic blood, diastolic blood, or pulse pressure). The authors also found an 18% overlap between kidney genes and CKD-defining traits, and characterized FDA-approved drugs and small chemical compounds on their ability to induce or reverse changes in gene expression related to high blood pressure. Of note, the model also provided insight into the transcriptome profile of cells harvested non-invasively from urine for the potential use as a diagnostic tool of kidney health and damage. 

Publicly available patient sample databases (cell/tissue repositories, atlases, etc.) are essential for developing effective computational models. Association studies (e.g., of the transcript, epigenome, proteome, metabolome, etc.) are critically important in model development and validation, especially the use of human samples to aid the translation of fundamental research findings, especially for drug development or preclinical trials. Other techniques to gather high-throughput datasets or computational models of the epigenetic landscape of HN remain to be developed, such as CRISPR modification and screens. These methods can provide additional insights or validation tools to identify and translate gene expression changes to phenotype and disease progression. Previous works using CRISPR genome modification and screening have already postulated such a connection between the kidney, its epigenome, and disease. For example, a gene network resource was published by Ungricht and colleagues in 2022 from their study of genome-wide CRISPR screens in iPS-cell-derived human kidney organoids that revealed temporal and cell-type-specific regulatory pathways associated with nephrogenesis and the association of several markers that relate to childhood nephrotic syndromes and ciliopathies [132]. Overall, for HN, computational modeling tools can help assign signature expression patterns with increased fidelity to the patient’s outcome compared to animal models, even when sample availability and size is limited; however, when combined with publicly available resources, the strategies outlined in this section can help build more accurate algorithms or predictive modeling tools that can address multiple unmet needs, including the discovery of new and effective disease biomarkers and the discovery of novel therapeutic targets.

## 4. Pathophysiology of HN

### 4.1. Hemodynamic Changes in Hypertension

#### 4.1.1. Arteries and Arterioles

Hypertension poses a significant hemodynamic burden on both large and small arteries and arterioles within the vascular system, including the renal arteries and the afferent and efferent arterioles in the kidney. In large arteries, high blood pressure accelerates the process of atherosclerotic renal artery stenosis, in which plaque builds up on the arterial walls, causing the thickening and hardening of the tissue [133]. In small arteries and arterioles, hypertension induces structural changes, such as hyaline arteriolosclerosis, characterized by the thickening of the vessel walls and narrowing of the lumen [134,135,136]. The high blood pressure damages the endothelial cells lining blood vessels, promoting inflammation and the accumulation of lipids and other substances, thereby leading to atherosclerotic plaque formation [137,138]. In both atherosclerosis and arteriosclerosis, the blood supply to the kidney has been shown to be limited due to the narrowing of blood vessels, leading to ischemia in various kidney tissues and generating more mechanical stress on the nephrons and their respective cell populations [139]. However, an opposing view suggests that atherosclerotic renal artery stenosis does not reduce renal oxygen levels [136]. Recently, Miyaoka et al. [140] demonstrated a strong correlation between increased renal arteriolar wall thickness and elevated central systolic blood pressure in patients with renal disease. In 2011, Briet et al. [141] further suggested that arterial remodeling and stiffening associated with CKD progression might arise from the excessive ECM turnover, the lack of vascular smooth muscle cell proliferation, or apoptosis. Other studies demonstrated that the activation of RAAS facilitates renal stenosis and can be targeted by ACE inhibitors to reduce the hypertensive condition in CKD [142,143].

#### 4.1.2. Peritubular Capillaries

Peritubular capillaries (PTCs) filter waste products from the blood into the tubular fluid and it also reabsorbs essential nutrients, such as amino acids, minerals, and glucose, from the glomerular filtrate back into the bloodstream [144]. Under hypertensive conditions, PTC rarefaction can occur, a condition characterized by the continuous loss of PTC density in the tissue due to endothelial cell apoptosis and pericyte detachment [145]. The loss of PTC density is associated with tissue hypoxia, further leading to tubulointerstitial fibrosis and a decrease in glomerular filtration function [146,147,148,149]. The underlying mechanisms of hypertension-induced PTC rarefaction indicate the importance of factors such as endothelial dysfunction, oxidative stress, inflammation, and the activation of the RAAS signaling [150,151,152,153].

Given these mechanisms, researchers have explored various approaches for detecting and mitigating PTC damage in hypertensive conditions. Iwazu et al. [154] demonstrated that the decrease in PTC density in DOCA salt-induced hypertension is accompanied by an increase in the expression of hypoxia responsive angiogenic factors VEGF-A and profibrotic growth factor TGF-β1. Recent therapeutics targeting RAAS have been shown to mitigate PTC damage in rat chronic intermittent hypoxia models. For instance, Wu et al. [155] demonstrated that losartan alleviates PTC loss by decreasing Ang II expression,. Similarly, Remuzzi et al. [156] showed that Ang II antagonists protect PTCs by normalizing TGF-β and Endothelin 1 (ET-1) gene expression levels. Endothelins and their receptors in the PTCs are also key regulators of endothelial function, mesangial cell proliferation, and vasoconstriction [157]. Targeting endothelin-A receptors has demonstrated protective effects against hypertension-induced vascular damage [158].

#### 4.1.3. Glomerular Capillaries

The glomerular capillaries, endothelial cell layer, the glomerular basement membrane, and specialized epithelial cells called podocytes together form the functional blood filtration apparatus. In hypertension, glomerular capillaries are especially vulnerable to injuries due to the sensitivity of their delicate filtration barrier in response to changes in mechanical stress [159]. Hyperfiltration, combined with inflammation induced by a hypertensive environment, damages the endothelial cells and podocytes, leading to the fibrotic remodeling of the glomerular basement membrane. This progressive damage leads to glomerulosclerosis and nephron loss, further compromising kidney function [160,161,162]. In addition to the glomerular capillary damage, failure to filter toxins can lead to secondary damages in the downstream renal tubules [163,164].

Endlich et al. [165] and Richfield et al. [166] used a mathematical model to calculate the mechanical stress experienced in glomerular capillaries during hypertension (50 kPa circumferential wall stress and 0.8 kPa shear wall stress) and its potential effects on the glomerular filtration barrier. The effect of the increased tensile stretch and shear stress on the filtration barrier is further illustrated by the observation that podocytes detach and die in hypertensive environments [167]. Hartner et al. [168] demonstrated that the α8 integrin expression in mesangial cells provides mechanical support to the glomerular capillary tuft, reducing glomerular capillary microaneurysm and the mesangiolytic lesion in hypertensive environments. Beyond mechanical stress, the molecular mechanisms contributing to glomerular capillary damage during hypertension are similar to those affecting the PTCs. These mechanisms involve key pathways, such as RAAS activation, glomerular endothelial dysfunction, and inflammation [169,170,171].

Hypertension induces both structural and functional damage in the renal vasculature, leading to atherosclerotic renal artery stenosis and hyaline arteriolosclerosis, which cause reduced blood flow and ischemia. Hypertension-induced damage extends to the PTCs, resulting in rarefaction and fibrosis, whereas, to the glomerular capillaries, HN causes hyperfiltration, inflammation, and glomerulosclerosis. The underlying mechanisms involve endothelial dysfunction, oxidative stress, inflammation, and RAAS activation, with potential therapeutic targets including ACE inhibitors and endothelin-A receptor antagonists to mitigate damage.

### 4.2. Hypertension and Glomerular Damage

#### 4.2.1. Glomerular Hypotrophy and Hyperfiltration

When the kidneys experience significant nephron loss due to chronic hypertension, compensation for the lost nephrons occurs through physical tissue expansion and increased hyperfiltration in the glomeruli of the remaining kidney, temporarily boosting filtration function [172,173,174]. One of the primary morphological changes observed in the glomeruli of hypertensive patients, as identified in biopsy samples, is glomerular hypertrophy [175]. Hill et al. reported that, compared to kidneys from normotensive patients, those from hypertensive patients show a 10% increase in hypertrophic and focal segmental glomerulosclerosis (FSGS)-type glomeruli. Additionally, within these hypertrophic glomeruli, the total capillary area was significantly larger compared to that in normotensive individuals (22,205 ± 10,426 μm^2^ vs. 15,349 ± 4577 μm^2^, *p* = 0.0038) [176]. The increase in glomerular size is correlated with the thickening of the glomerular basement membrane and expansion of the mesangial matrix [177,178]. Gene therapy with the delivery of the kallikrein gene into DS hypertensive rats with induced kidney disease led to a significant reduction in renal fibrosis, inflammation, and glomerular hypertrophy [179]. Both glomerular hypertrophy and sclerosis exacerbate stress and damage to the remaining nephrons, leading to further nephron loss. This creates a vicious cycle that exacerbates hypertension and perpetuates glomerular injury in HN [174,180].

#### 4.2.2. Glomerular Endothelial Cells

Glomerular endothelial cells of the vasculature are sensitive to hypertension and can be affected through several mechanisms, such as mechanical shear stress, oxidative stress, inflammation, and endothelial nitric oxide synthase (eNOS) dysfunction [181,182,183,184]. The glomerular endothelial cells lining the glomerulus are crucial for blood filtration and form a selective barrier along with podocytes and the basement membrane. Glomerular endothelial cells, with their unique fenestrated phenotype, allow the size-dependent selective restriction of protein passage [185,186]. Although evidence on the loss of fenestration phenotype in HN is limited, decreased levels have been documented in other CKD, such as diabetic nephropathy, where it is linked to glucose-induced oxidative stress, proinflammatory states, and mitochondrial dysfunction [186,187]. 

Beyond the fenestration phenotype, molecular mechanisms contributing to endothelial cell dysfunction in hypertension include a decreased KLF2 expression and the activation of NF-κB, which together exacerbate the transcription of genes associated with vascular dysfunction and inflammation [188]. Endothelial PAS domain protein 1 (EPAS1) deficiency leads to the activation of parietal epithelial cells, contributing to glomerular scarring and the development of FSGS [189]. Furthermore, glomerular endothelial dysfunction is a key factor in the development of glomerulosclerosis, exacerbating glomerular damage [190]. Studies by Luo et al. [191] and Chen et al. [192] have shown that endothelial damage in HN can be further aggravated by hypoxia-inducible factors (such as endothelial hypoxia-inducible factor-1α) and other inflammatory stressors (such as IL-6, CRP, and TNF-α), leading to the transcriptional activation of genes involved in inflammation and fibrosis and thereby promoting disease progression.

#### 4.2.3. Podocytes

Podocytes are specialized epithelial cells in the glomerulus of the kidney that form part of the filtration barrier by wrapping around the capillaries and forming interdigitations with their foot processes, achieving the selective filtration of molecules based on size and charge. In HN, mechanical stress compromises the structural and morphological integrity of podocytes, specifically causing foot processes’ effacement and the disintegration of the slit diaphragm, which ultimately impairs molecular filtration and the selectivity of the glomerular capillary wall. Affected podocytes undergo hypertrophy to compensate for the decreased foot process structure and loss of other podocytes, further jeopardizing the cell structure and eventually leading to detachment and apoptosis. Even in early stages of HN, the detachment of podocytes and effacement of their foot processes were detectable in patient urine samples.

Studies by Li et al. found that HN upregulates the expression level of LIM domain protein FHL2 in podocytes, which further mediate the Ang II-induced Rac1 activation. Knockout of the FHL2 protein does not affect normal kidneys but protects the podocyte foot processes from effacement in a hypertensive environment [193]. Research on the mechanosensitive channel Piezo1 indicates that the upregulation of Piezo1 in podocytes under hypertensive conditions contributes to Rac1 activation and cellular injury, which can be reversed by either Rac or Piezo1 inhibitors [194]. In a separate study, podocytes expressing the endogenous calpain inhibitor calpastatin prevented the Ang II-dependent inhibition of autophagy and hypertension-induced podocyte damage [195]. Uijl et al. [196] demonstrated that interventions targeting the RAAS can help mitigate podocyte injury and reduce albuminuria.

In conclusion, HN leads to compensatory mechanisms such as glomerular hypertrophy and hyperfiltration, which, although temporarily beneficial, ultimately exacerbate nephron loss and glomerular injury. The interplay of endothelial and podocyte dysfunction, including the loss of fenestrations, activation of inflammatory pathways, and structural damage to podocytes, accelerates the progression of kidney disease. These processes underscore the importance of therapeutic strategies targeting specific molecular pathways, such as the RAAS and mechanosensitive channels, like Piezo1, to mitigate renal damage and improve outcomes in hypertensive patients.

### 4.3. Hypertension and Renal Tubule Damage

The renal tubules function to reabsorb essential nutrients and water, secrete waste products and excess ions, regulate the electrolyte and acid–base balance, and concentrate urine, thereby maintaining the body’s fluid and electrolyte homeostasis [197]. Hypertension leads to significant damage in the renal tubules primarily through mechanisms such as ischemia, hypoxia, tubulointerstitial fibrosis, epithelial-to-mesenchymal transition (EMT), and oxidative stress.

#### 4.3.1. Ischemia and Hypoxia

One of the primary consequences of hypertension is the thickening and narrowing of renal arteries and arterioles, restricting the blood flow and oxygen supply, which leads to the ischemia and hypoxia of tubular cells, and, therefore, reducing the ability to filter waste and concentrate urine [198,199]. Øvrehus et al. found that early stages of HN, characterized by hypoxia and ischemia, result in disruptions in the serine/glycine and methionine/homocysteine metabolism. These metabolic disturbances may contribute to endothelial dysfunction, atherosclerosis, and renal fibrosis [200]. Lee et al. found that HN in SHRs leads to higher basal oxygen consumption rates and metabolic activity in renal tubular cells [201]. By targeting hypoxia in the renal tubules, Wu et al. showed that losartan downregulates the expression of Ang II and upregulates VEGF-A, rescuing the renal tubule structure in a hypoxic environment in chronic intermittent hypoxia rats [155]. Textor et al. [202] reported a correlation between an increased filtered sodium load and medullary hypoxia, underscoring the impact of hypertension on renal oxygenation and highlighting the increased risk of hypertension and kidney tissue injury during medullary hypoxia.

#### 4.3.2. Tubulointerstitial Fibrosis

Another major pathological feature of hypertension-induced renal tubule injury is tubulointerstitial fibrosis, characterized by the accumulation of the interstitial extracellular matrix, the infiltration of inflammatory cells, the loss of tubular cells, the proliferation of fibroblasts, and a reduction in the density of the PTC network [203,204]. Ang II is a well-known inducer of tubulointerstitial fibrosis. In Ang II-induced hypertensive rats, TGF-β and NLRP3 inflammasome activation were significantly upregulated, and the release of high-mobility group box 1 (HMGB1) caused fibrosis [205]. The depletion of NLRP3 reduces tubulointerstitial fibrosis by restoring mitochondrial dysfunction and alleviating ER stress [206]. The TGF-β/SMAD signaling pathway is implicated in kidney fibrosis [207]. In DOCA salt-induced HN, the G-protein-coupled receptor GPR97 is upregulated by hypertension. The depletion of GPR97 inhibits the TGF-β1/SMAD signaling pathway, consequently reducing tubulointerstitial fibrosis [208]. On the contrary, Tampe et al. detailed the progression of fibrosis in different renal compartments during HN, suggesting that diffuse fibrosis does not correlate with long-term renal injuries and emphasizing the complexity of this pathological process [209].

#### 4.3.3. Epithelial–Mesenchymal Transition

EMT describes a cellular process in which epithelial cells take on mesenchymal characteristics and behaviors and lose their original cell–cell junctions and polarized structure. In renal tubules, EMT of the renal tubule epithelial cells leads to myofibroblast differentiation and ECM accumulation [210], causing fibrosis and compromising the function of the renal tubules [211,212]. In Ang II-induced hypertensive rats, EMT is marked by an increase in the mesenchymal marker α-SMA and a decrease in the epithelial marker VE-cadherin. ET-1 facilitates EMT via the activation of the ROCK and yes-associated protein (YAP) mechanotransduction pathways, and the application of the ET-1 receptor antagonist bosentan halts the EMT process in HN [213]. The Ang II increase is a common characteristic in HN, and Yang et al. found that Ang II can facilitate EMT through its AT1 receptor, the activation of its downstream SMAD2/3 signaling pathway, and the inhibition of SMAD7. In 5/6 nephrectomy rats, SMAD7 inhibits the activation of SMAD2/3 and prevents tubular EMT [214]. The role of other RAAS pathways in HN-associated EMT is further supported by multiple studies [215,216]. In human biopsy samples from patients with HN, an RNA-seq analysis and immunohistochemistry (IHC) demonstrated the overexpression of genes associated with partial EMT, inflammation, and ECM remodeling, further supporting the role of HN-induced EMT in tubulointerstitial fibrosis [217].

#### 4.3.4. Oxidative Stress and Inflammation

Oxidative stress and inflammation are additional contributors to the damage of renal tissue in HN. Elevated levels of reactive oxygen species (ROS), such as free radicals and peroxides, in the kidneys contribute to vasoconstriction, increased renin release, the impaired function of glomerular cells, and proteinuria [218]. Oxidative stress also leads to inflammation and increases inflammatory cytokines in CKD [219], which can accelerate renal fibrosis and renal vascular injuries [220]. In the DOCA salt hypertension rat model, there was increased IL-18 expression, as a result of oxidative stress, on tubular epithelial cells accompanied by tubulointerstitial fibrosis; however, IL-18-/- mice experienced a significantly lower level of renal fibrosis and inflammation [221]. Hypertension also increases NF-κB activity and the expression levels of monocyte chemoattractant protein-1 (MCP1) and IL-1β in renal tubules. The knockdown of the actin-binding protein cofilin-1 disrupts the nuclear transportation of NF-κB, cutting off the inflammatory signaling pathway in hypertension [222]. Elmarakby et al. also demonstrated that the inhibition of the inflammatory cytokine TNF-α attenuates renal injury in DOCA salt hypertensive rats [223], and the inhibition of TNF-α and its related pathway has been shown to slow the progression of renal fibrosis [224].

#### 4.3.5. Ferroptosis

Ferroptosis is a regulated cell death mechanism driven by iron-induced lipid peroxidation, leading to oxidative damage and cell death [225]. Iron-dependent ferroptosis directly triggers the synchronized necrosis of renal tubules, as shown by intravital microscopy in models of ischemia-reperfusion injury (IRI) and oxalate crystal-induced acute kidney injury [226]. In the Dahl salt HN rat model, significantly higher levels of iron deposition was also observed in kidney tissues compare to normal rats using Prussian blue staining. In addition, TEM images revealed that HN rats have a significantly reduced mitochondrial volume and increased density of mitochondrial double membranes which are hallmarks of ferroptosis [227]. Ferroptosis is also a promising therapeutic target for HN: in Ang II- and 2K1C-induced mice HN models, the stimulator for interferon genes pathway is upregulated and directly interact with Acyl-CoA Synthetase Long-Chain Family Member 4 (ACSL4) which plays an essential role in initiating ferroptosis-related lipid metabolism and peroxidation. On the other hand, the siRNA inhibition of ACSL4 reduces hypertension-induced inflammation and fibrosis in renal tubules [228]. In 5/6 nephrectomy rats, ferroptosis inducer cisplatin exacerbated the decline in renal function and promoted inflammation and fibrosis in renal tubules, while the ferroptosis inhibitor deferoxamine mesylate inhibited the deposition of ECM proteins such as α-SMA and COL I, while lowering TGF-β1 induction [98].

#### 4.3.6. Other Pathways

Finally, the involvement of various signaling pathways, including TGF-β1 and the ubiquitin–proteasome system, as elucidated by Zhang et al. [229] and An et al. [230], underscores the complex molecular mechanisms underlying hypertensive renal damage. The therapeutic potential of targeting these pathways, such as through the use of fibroblast growth factor 21 (FGF21), has been explored by Weng et al. [231], where the authors reported a reduction in inflammation and oxidative stress in salt-sensitive, hypertension-induced nephropathy. In the autonomic nerve system, an increase in renal sympathetic nerve activity found in HN leads to reduced renal excretory function by affecting the renal vasculature, tubules, and juxtaglomerular granular cells [232]. Meanwhile, renal denervation has been shown to decrease the blood pressure in HN and restore kidney function [233].

In summary, hypertension causes multifaceted damage to the renal tubules that is driven by ischemia, fibrosis, EMT, oxidative stress, and inflammation. Understanding these mechanisms is crucial for developing effective therapeutic strategies to mitigate renal damage and preserve kidney function in hypertensive patients.

## 5. Epigenetics of HN: Synthesis and Prospects for Therapeutic Discovery

Extensive research has highlighted the involvement of various molecular pathways in different kidney diseases, and efforts to uncover commonalities among them remain ongoing. In this section, we propose that reactive oxygen species (ROS) play a critical role in the epigenetic regulation of kidney diseases, based on a comprehensive comparison of different kidney injury manifestations. Furthermore, we investigate key hypertension-associated signaling pathways, including the RAAS and EMT, which have shown significant links with ROS regulation. We emphasize the potential of epigenetic-based therapies targeting molecular mechanisms in HN, highlighting the prospects for therapeutic interventions.

### 5.1. Overview of the Role of Reactive Oxygen Species in Kidney Diseases

Reactive oxygen species are free radicals containing oxygen that easily react with other molecules in a cell. These species play an essential role in all types of kidney diseases by connecting various stimuli with multiple epigenetic modifications and downstream signaling pathways.

The accumulation of advanced glycation end products (AGEs) in hyperglycemic conditions is a fundamental abnormality associated with diabetic nephropathy [234]. As heterogeneous molecules, AGEs are derived from nonenzymatic reactions between glucose or other saccharide derivatives and proteins or lipids and can be induced by various environmental factors. The triggering of downstream signaling pathways by AGEs depends on their binding to receptors for AGE (RAGEs), which are multiligand receptors belonging to the immunoglobulin superfamily and are expressed on many types of cells [235]. The binding of AGEs to RAGEs promotes the downstream expression of the enzyme NADPH oxidase 2 (NOX2), leading to ROS production and NF-κB activation, which, in turn, promotes further RAGE expression [236]. 

Ang II, the primary vasoconstrictor of the RAAS, is involved in the induction of hypertension and has been shown to play an important role in increasing ROS [3]. Elevated levels of Ang II cause oxidative stress through AT1R and upregulate the renal cortical mRNA expression of p22phox and NOX1 [237]. Blocking AT1R with losartan in 5/6 nephrectomized rats also decreases ROS levels [238]. Another important signaling pathway that increases ROS involves adenosine binding to the adenosine type 1 receptor (A1R), which shares the same downstream pathway as AT1R via NOX and p38 mitogen-activated protein kinases (MAPKs) [239,240]. 

The relationship between ROS and kidney injuries has been observed not only in CKD but also in AKI. Meng et al. used apocynin as an ROS scavenger and observed the attenuation of cisplatin-induced renal function decline both in vitro and in vivo. The depletion of NOX4 led to renal function recovery, which suggests a role for NOX4 in ROS-mediated cell death and inflammation [241].

### 5.2. ROS in Epigenetic Modification

#### 5.2.1. ROS in DNA and Histone Modification

On DNA, ROS induce modifications that affect gene expression and genomic stability [242,243,244]. Extensive research has provided evidence that ROS affects DNA methylation by regulating the expression and activity of key enzymes involved in DNA methylation and demethylation. Researchers have found that ROS can promote or inhibit DNA methylation. For example, long-term exposure to hydrogen peroxide was shown to decrease the S-adenosyl methionine (SAM) level in HK2 cells while increasing glutathione levels, leading to the hypomethylation of long interspersed nuclear element-1 (LINE-1), which has recently been linked to CKD [245]. In contrast, Zhao et al. demonstrated that elevated ROS levels inhibits N-myc downstream-regulated gene 2 (NDRG2) by methylating its promoters through altering the DNMT expression in HK2 cells [246], indicating that ROS can also induce DNA hypermethylation. These findings suggest that both hypermethylation and hypomethylation induced by ROS can occur within the same cell line, highlighting a potentially significant research area for future investigations aimed at illuminating molecular mechanisms.

At the histone level, ROS have also been reported to modulate histone methylation marks, affecting both histone methyltransferases (HMTs) and histone demethylases (HDMs). Mentch et al. demonstrated that HMTs are regulated through the methionine metabolism pathway, which is also modulated by ROS [247]. Additionally, the inhibition of lysine-specific demethylase (LSD1), a member of HDMs, by polyphenols acting as ROS scavengers was observed in C2C12 fibroblasts [248], indicating that ROS regulate the expression and activity of HDMs. 

Histone acetylation and deacetylation are other major manifestations of histone modifications, and ROS can influence this process by modulating the expression and activity of HATs and HDACs. Increased hydrogen peroxide levels were shown to promote the recruitment of the HAT p300/CBP-associated factor (p300/CBP) to the matrix metallopeptidase 1 (MMP-1) promoter, increasing its expression [249]. Lazar et al. demonstrated that the activation of p300/CBP can enhance ROS production, creating a positive feedback loop in diabetic kidney disease models [250]. Conversely, ROS have been associated with decreased sirtuin 1 (SIRT1) activity in both cardiovascular diseases and kidney injuries [251,252]. For instance, the administration of resveratrol, a SIRT1 inhibitor, was found to prevent the progression of cardiac dysfunction and concentric hypertrophy in SHRs [253]. Huang et al. also demonstrated that the overexpression of SIRT1 in rat glomerular mesangial cells inhibits ROS production and fibrosis induced by AGEs [254].

In conclusion, there is strong evidence that ROS plays a pivotal role in regulating DNA methylation and histone modifications by altering the expression and activity of epigenetic enzymes. These epigenetic modifications are closely linked to renal injuries.

#### 5.2.2. ROS and Non-Coding RNA

As previously discussed, an increasing body of evidence suggests that non-coding RNAs, including miRNAs and lncRNAs, play a significant role in kidney diseases [255,256]. There is also evidence that non-coding RNAs are crucial regulators in response to ROS [257]. Among the various miRNAs regulated by ROS, several, such as miR-9 and miR-21, can also regulate ROS levels [258,259]. ROS can control miRNA gene expression indirectly through the regulation of DNA methylation and histone acetylation. For example, He et al. found that ROS increased the methylation of miR-199 and miR-125, thus upregulating hypoxia inducible factor subunit alpha (HIF1α) [260]. lncRNAs can also be regulated by ROS levels, as Wang et al. found that ROS upregulated lncRNA gadd7, resulting in ROS-induced endoplasmic reticulum (ER) stress and cell death [261]. Furthermore, oxidative stress has been shown to alter the expression of miRNAs in renal cells. For example, Muratsu-Ikeda et al. demonstrated that miR-205 plays a protective role against oxidative stress in HK2 cells via suppressing egl-9 family hypoxia inducible factor 2 (EGLN2), subsequently reducing ROS levels and presenting a potential therapeutic target in AKI and CKD [262].

Research exploring the relationships between ROS, non-coding RNAs, and CKD remains limited, and further studies are needed to elucidate these connections more comprehensively. Given the established role of ROS in kidney diseases and epigenetic modifications, it is plausible that the aforementioned ROS-related epigenetic pathways may also be involved in the progression of HN. The presence of similar or related epigenetic modifications across different types of renal cells suggests potential avenues for epigenetic-based therapies as discussed below.

### 5.3. Epigenetic Landscape of HN-Associated Signaling Pathways

As discussed earlier, key pathophysiological changes in HN include glomerular damage mediated by the RAAS and EMT following tubular cell injury under high blood pressure. A number of research studies have revealed molecular level connections and dependencies between these two signaling pathways and their relevance in epigenetic modulations.

#### 5.3.1. Epigenetic Regulation of RAAS

Angiotensinogen (AGT), present in blood vessels under normal conditions, is the substrate of renin and is converted to Angiotensin I (Ang I) when blood pressure drops. Ang I needs to be further converted to Ang II via catalyzation by the Angiotensin-converting enzyme (ACE) to initiate RAS signaling. ACE2 generates forms of angiotensin distinct from Ang II, thus competing with ACE1 to maintain the homeostasis of total ACE function [263]. Ang II interacts with AT1R and triggers the downstream ROS production and subsequent inflammation response. In this molecular network, epigenetic regulations occur in modulating the expression or activity of the AGT gene, ACE1/2, and AT1R. Human AGT expression was found to be inversely associated with the methylation status of the region near its promoter, indicating the regulation of AGT expression through DNA methylation [264]. Multiple miRNAs are also shown to influence AGT expression, such as miR-133a, miR-149-5P, and miR-29a [265,266,267]. The promoter of human ACE reportedly contain CpG islands for which methylation has been linked to hypertension in SHRs [268]. The histone modification of ACE was also observed by Lee et al. in the heart and kidney of SHRs with ACE upregulation [268]. Similarly, the AT1R gene is regulated by DNA methylation shown by Shan et al. recently as the hypermethylation of its promoter has been associated with reduced blood pressure in SHRs [269].

Although there is no direct research demonstrating how altering the epigenetic landscape of these aforementioned genes in RAAS could help prevent HN, the epigenetic regulation of RAAS has been observed in both SHRs and Munich Wistar Frömter (MWF) rats with an inborn nephron deficit and potential secondary hypertension [270,271], suggesting the involvement of epigenetic regulation in HN. Beyond RAAS-related genes, the downstream effects of RAAS activation on nephropathy-related genes also show epigenetic involvement. For example, Takahashi et al. found that blocking Ang II receptor increases nephrin expression in rat puromycin aminonucleoside nephropathy and ameliorates proteinuria [272]. Furthermore, Hayashi et al. demonstrated that using ARBs such as candesartan and irbesartan reduces the methylation of the nephrin promoter with the recovery of KLF4 expression and a decrease in albuminuria [27]. Another example is that transient receptor potential canonical type 6 (TRPC6), a protein associated with proteinuria when aberrantly overexpressed, has been shown to be upregulated by Ang II [273,274]. The underlying epigenetic mechanism of TRPC6 regulation by Ang II was further shown by Zhao et al., where the authors indicated that Ang II upregulates TRPC6 expression by downregulating miR-30a [275]. Collectively, these studies indicate that several molecules within the RAAS pathway are controlled by epigenetic mechanisms, offering potential therapeutic targets. However, further research is needed to explore the full extent of epigenetic regulation in common signaling pathways within the RAAS regulatory network.

#### 5.3.2. Epigenetic Regulation of EMT

Rodrigues-Díez et al. have demonstrated that EMT is also regulated by Ang II through MAPKs and RhoA/ROCK signaling pathways in HK2 cells [276]. The authors showed that inhibiting MAPKs and Rho-kinase (ROCK) with specific inhibitors downregulate CTGF and prevent EMT caused by Ang II, highlighting the signaling transduction in Ang II-induced EMT signaling pathway [276]. Researchers have since explored multiple signaling pathways related to EMT progression. In addition to MAPK/ERK and RhoA/ROCK pathways, the TGF-β/SMAD2 signaling pathway has also been implicated in EMT regulation. Carvajal et al. found that Ang II activates SMAD signaling by TGF-β-mediated processes and causes EMT [277]. Further investigation into the epigenetic regulation of RhoA/ROCK and TGF-β/SMAD2 pathways revealed significant findings. With regard to RhoA/ROCK signaling, researchers found that PHF8, a histone demethylase, removes H3K9me on the promoter of ROCK kinase to promote gene expression and cell invasion in prostate cancer cells [278]. No studies have yet demonstrated the epigenetic regulation of ROCK in renal cells and this remains a potential direction for kidney diseases research. Compared with the epigenetic regulation of the ROCK pathway, there are more research studies reporting the involvement of the TGF-β/SMAD2 signaling pathway, where Papageorgis et al. showed that the recruitment of DNMT1 promotes EMT in breast cancer cells [279]. Similarly, Choi et al. found that hypertensive rats treated with HDAC6-specific inhibitors exhibited lowered TGF-β expression, suppressed histone acetylation, and reduced renal fibrosis [280]. In terms of non-coding RNA regulation, miR-200 family members have been shown to repress the expression of TGF-β2 and β-catenin to attenuate EMT [281,282]. These studies collectively highlight that EMT is regulated by diverse epigenetic mechanisms impacting several signaling pathways such as RhoA/ROCK and TGF-β/SMAD2. These pathways, influenced by DNA methylation, histone modifications, and non-coding RNAs, play a significant role in the progression of EMT. Given their involvement, these epigenetic modulations offer substantial potential for therapeutic intervention. Understanding how these mechanisms contribute to the pathophysiology of EMT can open new avenues for the development of targeted therapies aimed at multiple kidney diseases.

While research specifically linking epigenetic regulation to HN is still limited, there is a growing body of work demonstrating the epigenetic regulation of specific signaling pathways in other cell types or kidney diseases. These signaling pathways, which likely play an important role in the development of HN, may serve as potential therapeutic targets for HN treatment.

### 5.4. Therapeutic Targeting of Epigenetic Regulators

Following the growing research interest in the field of epigenetics, many drugs have been developed to alter epigenetic modifications implicated in disease. These drugs can generally be divided into three categories based on the function of their molecular targets: (1) “epigenetic writers” such as DNMTs, HMTs, and HATs; (2) “epigenetic erasers” such as TETs, HDMs, and HDACs; and (3) “epigenetic readers”, which are effector proteins that can specifically bind to certain types of epigenetic modifications to regulate gene expression. Researchers have found various inhibitors targeting each of these epigenetic modifiers, and some of them have already shown promise in treating CKD.

#### 5.4.1. Drugs Targeting Epigenetic Writers

Azacitidine (5′-azacytidine) is a DNMT inhibitor (DNMTi) approved by the FDA in 2004 for the treatment of myelodysplastic syndrome [283]. Another DNMTi, decitabine, which targets the same disease, was approved in 2006 [284]. These two drugs were later tested in kidney disease models and demonstrated renal protective effects by restoring the expression of downregulated genes involved in CKD progression, such as Klotho, RASAL1, and erythropoietin [285,286,287]. Additional information on these inhibitors of epigenetic writers can be found in Table 1.

#### 5.4.2. Drugs Targeting Epigenetic Erasers

Vorinostat, a histone deacetylase inhibitor (HDACi), received FDA approval in 2006 for treating cutaneous T-cell lymphoma [297]. This drug has also been used as a treatment for diabetic mice as its administration results in a reduction in albuminuria and fibrosis as well as decreased expression of inflammatory cytokines in splenocytes from MRL^lpr/lpr^ mice [288,298,299]. Valproic acid, originally used to treat epilepsy and granted early FDA approval, was later identified as an HDAC inhibitor. Research has shown that valproic acid can prevent histone demethylation, reduce renal injury in diabetic rats, and prevent proteinuria in ADR-induced nephropathy [289,300,301]. Hydralazine, an older drug approved by the FDA in 1953 for hypertension, was found to demethylate the RASAL1 promoter and attenuate renal fibrosis in preclinical models [292,302]. Hydralazine may also play a role in upregulating TET3 expression [275]. These inhibitors of epigenetic erasers are listed in Table 1.

#### 5.4.3. Drugs Targeting Epigenetic Readers

In addition to the aforementioned FDA-approved drugs, researchers have developed various inhibitors targeting epigenetic biomarkers. For example, the inhibiting methylation of H3K4 or H3K9 with sinefungin or BIX01294 has been reported to attenuate renal fibrosis in unilateral ureteral obstruction (UUO) mice [294,295]. Bromodomain and extra-terminal (BET) family proteins, which act as histone acetylation readers, bind to acetylated histone and participate in constructing transcriptional regulator complexes. MS417, a BET inhibitor, has been shown to reduce experimental diabetic proteinuria and kidney injury [296]. See Table 1 for additional information on these representative inhibitors and other relevant drug candidates.

### 5.5. Potential Epigenetic Therapies for HN

As mentioned in previous sections, the development and progression of multiple kidney diseases correlate with several epigenetic modifications regulated by DNMTs, HDACs, and other enzymes. Inhibitors of these epigenetic modifiers, such as 5′-azacytidine, decitabine, vorinostat, and valproic acid, exhibit renal protective effects in different kidney disease models. Given the commonalities between HN and other kidney diseases, including ROS-induced epigenetic regulations, it is conceivable that the progression of HN may also be attenuated by these epigenetic inhibitors. Nevertheless, additional work is needed to improve the specificity of these drug candidates; due to the widespread distribution of epigenetic modifications across tissues and organs in vivo, these drugs often lack precision and selectivity, which can lead to off-target effects and undesired side effects [303,304]. One way to address this issue could involve strategies to specifically target gene loci. Such an approach enables the accurate regulation of genes within specific regions with a level of precision needed for the development of effective therapeutic approaches for epigenetic modification. 

CRISPR-Cas-based genome editing, a technique that has gained popularity due to its high selectivity and specificity, is suitable for more accurate epigenetic modifications. The CRISPR/dCas9 system is a practical tool for epigenetic editing and utilizes the dCas9 protein, which lacks nucleolytic activity but retains binding efficiency to the target genome under the guidance of RNA [305]. To use dCas9 for epigenetic editing, an epigenetic mark-modifying domain is fused with the dCas9 protein. This complex can target specific genome loci in association with guide RNA, thereby conducting epigenome editing through the fused effectors [306]. 

For efficient epigenetic modifications, various effector proteins have been fused with dCas9. In one design, DNMT3A was fused with dCas9 to form the dCas9-DNMT3A complex, which suppresses gene expression by increasing methylation in the promoter region [307]. Conversely, Liu et al. designed dCas9-TET1 to achieve DNA demethylation in the promoter region of brain-derived neurotrophic factor (BDNF) [308]. Other effector protein designs include dCas9-HDAC3, dCas9-LSD1, and dCas9-EZH2 [309,310,311]. Based on this fundamental work, researchers have developed CRISPR/dCas9-based epigenetic modification tools targeting kidney injuries. For example, Xu et al. utilized high-fidelity TET-conjugated Cas9 to alter the expression of RASAL1 and Klotho in mice, demonstrating the protective effect of demethylating these two genes in attenuating kidney fibrosis [304]. This research suggests that the CRISPR epigenome editing system has the potential to address the limitations of existing small molecule drug-based epigenetic therapy.

Currently, the gold standard for managing hypertension and HN still involves using inhibitors of the RAAS. While these drugs are effective in treating hypertensive patients with chronic kidney diseases, limitations exist along with potential severe side effects due to non-specific targeting [5,6,7,8]. The CRISPR-based epigenome editing tool has already shown initial success in kidney injury therapeutics [304], indicating significant potential for application in precision medicine.

### 5.6. Exploring Epigenetic Signatures as Diagnostics for HN

Current clinical approaches for diagnosing HN include the following criteria: (i) primary and sustained hypertension; (ii) persistent proteinuria defined as urinary protein excretion over 150 mg per 24 h or an albumin-to-creatinine ratio exceeding 15 mg/mmol—considered a simpler and more reliable approximation of 24 h protein excretion; and (iii) the exclusion of various primary and secondary renal diseases [312,313]. Blood and urine are common samples used to evaluate kidney function. Although these analyses are efficient for diagnosing patients with abnormal clinical manifestations or routine testing results, they are unable to detect renal damage at the early stages of HN [314]. By the time HN is diagnosed through blood and urine tests, significant renal damage may have already occurred. Therefore, the early-stage diagnosis of HN is still needed in order to inform treatment strategies and help minimize hypertension-induced renal damage. This necessitates a precise and sensitive technique capable of detecting markers associated with the occurrence and development of HN.

Given its high specificity and sensitivity, the CRISPR-Cas system has become a highly valuable tool for detecting trace levels of molecules in biological samples. Leveraging this advantage, researchers have developed various detection techniques by combining CRISPR-based target locating and cleavage with multiple amplification and characterization methods. Specific high-sensitivity enzymatic reporter unlocking (SHERLOCK) [315], SHERLOCKv2 [316], DNA endonuclease-targeted CRISPR trans reporter (DETECTR) [317], and one-hour low-cost multipurpose highly efficient system (HOLMES) [318] are all rising techniques that show promise in rapidly detecting DNA and RNA viruses, discriminating single nucleotide polymorphisms (SNPs), and genotyping different strains of viruses. Considering the need for the early diagnosis of HN, highly sensitive CRISPR-based detection techniques could help diagnose HN as early as possible and prevent its deterioration via timely intervention.

As discussed in previous sections, epigenetic markers can serve as potential indicators of HN onset or progression. However, all the aforementioned assays (SHERLOCK, DETECTR, and HOLMES) are not efficient for detecting the epigenetic landscape of HN due to their inability to retain epigenetic modifications during amplification. To overcome these limitations, researchers have optimized CRISPR diagnostic system to detect epigenetic markers. For example, HOLMESv2 is an updated system capable of accurately quantitating target DNA methylation levels by combining Cas12b detection with bisulfite treatment [319]. Nanopore sequencing is another technique used to detect and quantify DNA methylation [320]. In addition to DNA methylation, non-coding RNA can also be detected using a nanozyme-catalysed CRISPR assay via a preamplification-free procedure [321]. These emerging techniques have not yet been applied to epigenetic diagnostics for HN, but they hold great potential for detecting candidate HN epigenetic markers, such as RAS protein activator like 1 (RASAL1) and Klotho hypermethylation, in small blood samples at the early stages of HN.

## 6. Conclusions

In conclusion, HN involves complex and multifaceted mechanisms that lead to significant kidney damage, including glomerular hypertrophy, endothelial and podocyte dysfunction, and renal tubule injury. HN-related kidney injury also further escalates the primary hypertension condition. This review article highlighted the critical roles of epigenetic modifications, such as DNA methylation, histone modifications, and non-coding RNAs, in the pathogenesis of HN. The interplay between these epigenetic mechanisms and key signaling pathways, such as the renin–angiotensin system and epithelial–mesenchymal transition, underscores the potential for targeted therapeutic interventions. Future research focused on understanding these epigenetic landscapes could pave the way for novel treatments aimed at mitigating renal damage and improving outcomes for patients with HN.

## Figures and Tables

**Table 1:** Representative compounds modulating epigenetic modifications in preclinical kidney disease models. * drugs with approval from FDA; UUO, unilateral ureteral obstruction; H3K4, histone 3 lysine 4; H3K9, histone 3 lysine 9.

Drug Name	Drug Target and Epigenetic Modification	Disease Model	Preclinical Result
5′-azacytidine *	DNMT inhibitor	Folic-acid-induced nephropathy, UUO	Attenuation of anemia, decreased renal fibrosis [285]
Vorinostat *	HDAC inhibitor	Diabetic nephropathy	Increased renal function, decreased renal oxidative stress [288]
Valproic acid *	HDAC inhibitor	Diabetic nephropathy	Decreased albuminuria, decreased glomerulosclerosis [289]
Resveratrol	SIRT1 inhibitor	Diabetic nephropathy	Increased renal function, decreased renal oxidative stress and inflammation [290,291]
Hydralazine *	Induces TET-dependent demethylation	IRI, obesity-induced CKD	Increased renal function, decreased fibrosis, decreased albuminuria [292,293]
Sinefungin	H3K4 methyltransferase SET7/9 inhibitor	UUO	Decreased fibrosis [294]
BIX01294	H3K9 methyltransferase G9a inhibitor	UUO	Decreased fibrosis [295]
MS417	BET inhibitor	Diabetic nephropathy	Decreased albuminuria [296]
